# Certified Nurse‐Midwife Practice Authority as a Structural Determinant of Maternal Health

**DOI:** 10.1111/1468-0009.70131

**Published:** 2026-09-17

**Authors:** CURISA M. TUCKER, JENNIFER BAUMSTARK

**Affiliations:** ^1^ Biobehavioral Health & Nursing Science College of Nursing University of South Carolina; ^2^ Advanced Professional Nursing Practice and Leadership College of Nursing University of South Carolina

**Keywords:** nurse‐midwives, scope of practice, workforce, maternal health, health policy, mediation analysis, health status disparities, vulnerable populations

## Abstract

**Policy Points**

- State scope‐of‐practice restrictions on certified nurse‐midwives are associated with lower workforce density and higher maternal vulnerability. In fact, 25 states currently impose collaborative or restricted practice environments that coincide with the nation's worst maternal health indicators.
- Mediation analysis revealed that 27% of the regulatory effect operates through workforce supply, whereas 73% reflects a direct policy effect, indicating that both regulatory reform and workforce investment are needed.
- Federal incentives through the Centers for Medicare and Medicaid Services's Rural Health Transformation Program now award scoring points based on state practice authority classifications, directly linking the state regulatory environment to federal funding and providing states with both an evidence‐based rationale and a financial incentive for reform.

**Context:**

Certified nurse‐midwives (CNMs) provide evidence‐based maternity care associated with improved birth outcomes, yet half of US states do not grant them independent practice authority. State scope‐of‐practice regulations may function as structural determinants of maternal health by constraining both the midwifery workforce and the conditions under which care is delivered. Prior studies have documented associations between midwifery regulation and workforce availability but have not formally tested the mechanisms linking regulation to population‐level maternal health outcomes.

**Methods:**

We conducted a cross‐sectional ecological analysis of all 50 US states, classifying each by CNM practice authority (independent, collaborative, or restricted) using American College of Nurse‐Midwives state practice environment data (March 2026). CNM workforce density was calculated from American Midwifery Certification Board data (2022‐2023). The primary outcome was the maternal vulnerability index (MVI), a composite of 43 indicators excluding provider density. The secondary outcome was March of Dimes preterm birth report card grades. We used one‐way analysis of variance, multiple linear regression, and Baron and Kenny mediation analysis with Sobel tests and bias‐corrected bootstrap confidence intervals to test whether workforce density mediated the relationship between practice authority and maternal vulnerability.

**Findings:**

States allowing independent practice had significantly higher CNM densities than collaborative states (6.5 vs. 2.9 per 1,000 births, *P* < .001), as well as lower MVI scores (2.1 vs. 4.1, *P* < .001). The effect sizes were large (Cohen's *d* = 1.34 for CNM density, *d* = −1.62 for MVI). CNM density was found to mediate 26.7% of the practice authority–MVI association (Sobel *z* = −2.10, *P* = .036; 95% bias‐corrected and accelerated [BCa] bootstrap CI [−0.88, −0.08]). The remaining 73.3% represented a direct policy effect that is not explained by workforce numbers. These results were robust to alternative classifications using nurse practitioner regulatory data.

**Conclusions:**

State CNM scope‐of‐practice regulations are a modifiable policy lever associated with both clinician workforce distribution and population‐level maternal vulnerability. Both regulatory reform and complementary workforce investments are needed to address maternal health inequities.

Maternal morbidity and mortality remain critical public health challenges in the United States. More than 80% of pregnancy‐related deaths have been deemed preventable by state and local maternal mortality review committees,^1^ yet the national maternal mortality rate reached 17.9 per 100,000 live births in 2024.^2^ Outcomes are profoundly inequitable: Black birthing individuals face three to four times the risk of pregnancy‐related death compared to White individuals,^2^ and rural communities experience elevated risk because maternity care is either limited or nonexistent.^3^ More than one‐third of US counties are classified as maternity care deserts, with no hospital offering obstetric services and no obstetric clinician.^4^

Evidence supports several approaches to improving maternal outcomes, including expanded access to midwifery care. A Cochrane systematic review found that midwife continuity of care models were associated with lower rates of cesarean birth and instrumental delivery and with higher rates of spontaneous vaginal birth and more positive patient experiences.^5^ US studies have demonstrated that women receiving prenatal care from midwives experience fewer cesarean births, lower rates of preterm delivery, and fewer labor interventions compared to women receiving physician‐led care.^6^, ^7^, ^8^ A recent scoping review of certified nurse‐midwife (CNM) and certified midwife (CM) care in the United States confirmed these findings across six patient care domains, reinforcing the breadth and quality of midwifery outcomes.^9^ The American College of Obstetricians and Gynecologists and the American College of Nurse‐Midwives (ACNM) jointly recognize CNMs as educated, trained, and licensed independent clinicians who are experts in their field.^10^ CNMs are educated in programs accredited by the Accreditation Commission for Midwifery Education, and upon graduation, they must demonstrate competency consistent with or exceeding the global standards for midwifery practice defined by the International Confederation of Midwives.^11^ This standardized preparation means that any state‐imposed limitation on CNM practice restricts care that a midwife is trained and certified to provide. Despite this evidence and interprofessional support, half of US states do not allow CNMs to practice to the full extent of their education and training.

This study focuses on CNMs because the practice authority data and workforce density data used in our analyses reflect the CNM regulatory and certification environment specifically. CMs share identical ACNM core competencies, sit for the same American Midwifery Certification Board (AMCB) certification examination, and have the same scope of practice as CNMs, but CMs differ in that they are not also licensed as nurses.^11^ CM licensure is currently recognized in only 12 states and the District of Columbia, and CMs represent a small fraction of the midwifery workforce. Although CMs face similar regulatory challenges, the available state‐level data do not permit separate analysis of CM practice patterns and outcomes. Certified professional midwives, who are certified through a separate pathway outside the ACNM/AMCB system, are not included in this analysis.

State scope‐of‐practice laws determine whether CNMs can evaluate patients, diagnose conditions, initiate treatments, and prescribe medications, including controlled substances, independently, or whether they must operate under physician supervision or collaborative agreements.^11^ The ACNM classifies state practice environments for CNMs into four categories: (1) independent practice, in which CNMs practice and prescribe autonomously; (2) collaborative agreement, in which a signed agreement with a physician is required as a condition of practice; (3) hybrid, in which CNMs practice independently for most services but face restrictions on a subset, such as prescriptive authority or intrapartum care; and (4) supervision, in which physician oversight of CNM practice is required.^12^ As of March 2026, 25 states and the District of Columbia granted independent practice authority, 17 states required collaborative agreements, 6 states maintained hybrid environments, and 2 states required physician supervision.^13^

These regulatory differences may function as structural determinants of health, which are defined as institutional and system‐level conditions, including laws, policies, and political structures, that shape the distribution of resources and opportunities and thereby influence health outcomes.^14^ Crear‐Perry and colleagues have established a framework positioning inequitable policies and systems, rather than the characteristics of the populations they serve, as the root drivers of maternal health disparities.^14^ CNMs are independent care providers during pregnancy, childbirth, and the postpartum period; beyond pregnancy‐related care, they provide family planning, preconception, reproductive, gynecologic, and sexual health care across the life span.^11^ Twenty‐five states currently impose collaborative, hybrid, or supervision requirements that restrict the ability of CNMs to provide this full scope of care.^13^ Scope‐of‐practice laws represent one such modifiable structural condition: they are enacted at the state level, they determine which clinicians can provide care and under what conditions, and they shape the geographic distribution of the maternity care workforce.

Several prior studies have examined the relationship between midwifery workforce regulations and outcomes. Yang and colleagues found that broader CNM scope of practice was associated with higher rates of CNM‐attended births and fewer cesarean deliveries.^15^ Markowitz and colleagues used quasi‐experimental methods to examine the competitive effects of scope‐of‐practice restrictions on the CNM workforce and related maternal and infant outcomes.^16^ Ranchoff and Declercq found that states with a broader scope of midwifery practice had significantly higher availability of certified nurse‐midwife–attended births.^17^ Herndon and Vanderlaan demonstrated associations between state practice regulations and access to midwifery care.^18^ In addition, Vanderlaan showed that workforce density moderated the association between independent practice and pregnancy outcomes, suggesting that regulatory effects are partly conditional on the number of clinicians available to benefit from expanded authority.^19^ The World Health Organization (WHO) recommends a minimum of six midwives per 1,000 births to ensure adequate midwifery coverage and safe maternal care.^20^ Vedam and colleagues developed a Midwifery Integration Scoring System and found that states with higher integration scores had fewer neonatal complications, higher rates of physiologic birth, and fewer interventions.^21^ However, these studies relied on bivariate, descriptive, or moderation‐based approaches and did not formally test the mechanism through which regulation affects outcomes, whether by constraining workforce supply or through direct effects on care delivery.

This study examines whether state‐level scope‐of‐practice regulations for CNMs are associated with clinician workforce density and population‐level maternal vulnerability and formally tests whether workforce density mediates this relationship using the Baron and Kenny causal steps framework.^22^ The maternal vulnerability index (MVI),^23^ a composite measure of 43 indicators of maternal morbidity and mortality risk, serves as the primary outcome. March of Dimes preterm birth report card grades^24^ serve as a secondary outcome.

## Methods

### Study Design and Data Sources

We conducted a cross‐sectional ecological analysis using aggregate state‐level data, rather than individual‐level patient records, for all 50 US states. An ecological study examines associations between exposures and outcomes measured at the group or population level rather than the individual level.^25^ The District of Columbia was included in descriptive summaries but excluded from analytic models because of its unique non‐state governance structure. State‐level data were compiled from four publicly available sources.

Practice authority classifications were obtained from the American College of Nurse‐Midwives state practice environment map for certified nurse‐midwives, updated in March 2026.^13^ The ACNM classifies CNM‐specific practice environments, which may differ from the broader nurse practitioner regulatory environment captured by the American Association of Nurse Practitioners. The ACNM map identifies four categories: independent practice, collaborative agreement, hybrid (independent for most services with restrictions on a subset), and supervision. For analysis, we collapsed these categories into three groups: independent practice (25 states), collaborative agreement (17 states), and restricted practice (8 states combining hybrid and supervision environments), as both hybrid and supervision categories involve mandatory limitations on CNM autonomy beyond those imposed by a collaborative agreement.

CNM workforce density data, expressed as the number of CNMs per 1,000 live births in each state, were obtained from Vanderlaan's State Midwifery Information Sheets, which calculated densities from 2022‐2023 AMCB certification data and 2022 state birth records.^26^ March of Dimes (MOD) preterm birth report card grades (A through F) were obtained from the 2025 March of Dimes Report Card for United States.^24^ MOD grades incorporate multiple components including preterm birth rates, access to care, and maternity care provider availability; because provider density is one component of these grades, analyses using the MOD grade as an outcome with CNM density as a mediator are subject to partial circularity. We therefore designated the MOD grade as a secondary outcome.

Maternal vulnerability index (MVI) scores were obtained from the Surgo Health MVI interactive database,^27^ which has been described and validated elsewhere.^23^ The MVI integrates 43 indicators of maternal morbidity and mortality risk, drawn from a scoping review of US studies published between 2000 and 2020, grouped into six thematic domains: reproductive health, physical health, mental health and substance abuse, general health care, socioeconomic determinants, and physical environment. State‐level MVI classifications (from very low through very high) were extracted from the database's state‐level view, which assigns an overall vulnerability category to each state based on aggregated county‐level indicator data.^23^ We used the 2025 MVI data. The MVI has been validated against adverse pregnancy outcomes including preterm birth and low birth weight.^23^ The MVI does not include provider density or workforce measures among its 43 indicators, making it suitable as the primary outcome in a mediation model where CNM density serves as the mediator.

### Variables

The independent variable was the state practice authority category (independent, collaborative, or restricted). The proposed mediator was CNM density per 1,000 live births. The primary dependent variable was the MVI score, converted into an ordinal scale as follows:1 = very low vulnerability, 2 = low vulnerability, 3 = moderate vulnerability, 4 = high vulnerability, 5 = very high vulnerability. The secondary dependent variable was the MOD preterm birth report card grade, which was also converted to a numeric scale (A = 5.0, A^−^ = 4.7, B^+^ = 4.3, B = 4.0, B^−^ = 3.7, C^+^ = 3.3, C = 3.0, C^−^ = 2.7, D^+^ = 2.3, D = 2.0, D^−^ = 1.7, F = 0). Although the MVI scores were derived from ordinal categories (1–5), they were treated as continuous in regression and mediation analyses, consistent with standard practice for ordinal scales with evenly spaced categories. Shapiro‐Wilk tests indicated departure from normality in the residuals (*P* < .001), which motivated the use of nonparametric Kruskal‐Wallis tests alongside analysis of variance (ANOVA) and bias‐corrected bootstrap confidence intervals for indirect effects, both of which confirmed the parametric results.

### Statistical Analysis

We performed one‐way ANOVA to compare CNM densities, MVI scores, and MOD grades across the three practice authority categories. Post hoc pairwise comparisons used Tukey's honestly significant difference (HSD) test with 95% confidence intervals. Pearson and Spearman rank correlation coefficients were used to assess bivariate associations. Effect sizes were computed using Cohen's *d* (independent vs. nonindependent practice authority) and eta squared for ANOVA models.

Multiple linear regression models examined the simultaneous effects of practice authority and CNM density on each outcome. We estimated five models: (1) practice authority predicting CNM density, (2) practice authority predicting MVI (total effect), (3) practice authority plus CNM density predicting MVI (mediation model), (4) practice authority predicting MOD grade, and (5) practice authority plus CNM density predicting MOD grade. Full regression specifications including all coefficients, standard errors, *t* statistics, *R*‐squared values, *F* statistics, and number of observations are provided in Appendix Table A1.

To test mediation, we applied the Baron and Kenny causal steps approach^22^ supplemented by the Sobel test for indirect effects. For mediation analysis, practice authority was dichotomized (independent vs. nonindependent) to yield a single coefficient for each path. This pooling was supported by post hoc Tukey HSD comparisons showing no significant differences between collaborative and restricted states for any outcome (all *P* > .05). We acknowledge that this grouping decision was informed by the data rather than prespecified. Path a estimates the effect of practice authority on the mediator (CNM density); path b estimates the effect of the mediator on the outcome, adjusted for practice authority; path c is the total effect; and path c′ is the direct effect. The indirect effect (a × b) represents the portion of the total effect transmitted through the mediator. To address concerns about the normality assumption underlying the Sobel test with a sample of 50 states, we supplemented the mediation analysis with bias‐corrected and accelerated bootstrap confidence intervals using 5,000 resamples. A *P* value of less than .05 was considered statistically significant. All analyses were conducted in R version 4.6.0. The study used publicly available aggregate data and was exempt from institutional review board review.

As a sensitivity analysis, we repeated all analyses using American Association of Nurse Practitioners (AANP) practice authority classifications, which reflect the broader nurse practitioner regulatory environment, to assess robustness to alternative regulatory data sources.

## Results

### Descriptive Findings

Table 1 summarizes descriptive statistics and group comparisons by practice authority category. Across 50 states, CNM density ranged from 0.6 per 1,000 live births (Alabama) to 16.6 per 1,000 live births (Vermont). The mean CNM density in states allowing independent practice authority was 6.5 (SD = 3.4), compared to 2.9 (SD = 1.4) in states requiring collaborative practice and 3.3 (SD = 1.0) in restricted states. Only 12 states met the WHO recommendation of at least six midwives per 1,000 births^20^; all 12 (100%) allowed independent practice authority.

**Table 1:** Certified Nurse‐Midwife Workforce Density and Maternal Health Indicators, by State Practice Authority Category, 50 US States, 2022‐2026

Variable	Independent^a^ (*n* = 25)	Collaborative^a^ (*n* = 17)	Restricted^a^ (*n* = 8)	*F* or *H* ^b^	*P* Value	η^2 ^ ^b^
**CNM Density**						
Mean ± SD	6.45 ± 3.37	2.87 ± 1.44	3.32 ± 1.01	*F* = 11.15	<.001	0.322
Median [IQR]	5.6 [4.0, 8.7]	2.8 [1.5, 3.9]	3.5 [2.4, 4.3]			
Range	2.2–16.6	0.6–5.0	2.0–4.3			
States above the WHO target,^c^ No. (%)	12 (48%)	0 (0%)	0 (0%)			
**MVI Score** ^d^ **(Primary)**						
Mean ± SD	2.12 ± 1.09	4.12 ± 1.05	3.62 ± 1.41	*F* = 17.01	<.001	0.420
Median [IQR]	2.0 [1.0, 3.0]	4.0 [4.0, 5.0]	4.0 [2.0, 5.0]	*H* = 20.73	<.001	
States with VH or H vulnerability, No. (%)	3 (12%)	13 (76%)	5 (62%)			
**MOD Grade** ^d^ **(Secondary)**						
Mean ± SD	3.17 ± 0.68	1.66 ± 1.17	1.59 ± 1.42	*F* = 14.73	<.001	0.385
Median [IQR]	3.0 [3.0, 3.7]	2.0 [0.0, 2.3]	2.0 [0.0, 2.4]			
**Cohen's *d*** ^d^ **(Independent vs. Nonindependent)**						
CNM density	1.34					Large
MVI score	−1.62					Large
MOD grade	1.55					Large

ANOVA, analysis of variance; CNM, certified nurse‐midwife; H, high; IQR, interquartile range; MOD, March of Dimes; MVI, maternal vulnerability index; SD, standard deviation; VH, very high; WHO, World Health Organization.

^a^ Independent means autonomous CNM practice; collaborative means collaborative agreement required; restricted means hybrid restrictions or physician supervision required.

^b^ *F*, one‐way ANOVA *F* statistic (df = 2, 47); *H*, Kruskal‐Wallis statistic; η^2^, eta‐squared effect size.

^c^ The WHO target is at least 6 midwives per 1,000 births.

^d^ MVI scores were classified as follows: 1 = very low vulnerability, 2 = low vulnerability, 3 = moderate vulnerability, 4 = high vulnerability, 5 = very high vulnerability. MOD grades were converted to a numeric scale (A = 5 to F = 0). Cohen's *d* was computed for states with independent (*n* = 25) vs. nonindependent (*n* = 25) practice authority.

Data were obtained from the American College of Nurse‐Midwives state practice environment map (March 2026),^13^ American Midwifery Certification Board workforce data (2022‐2023) via Vanderlaan (2025),^26^ the Surgo Health US maternal vulnerability index map (2025),^27^ and the 2025 March of Dimes Report Card for United States.^24^

**Figure 1:**
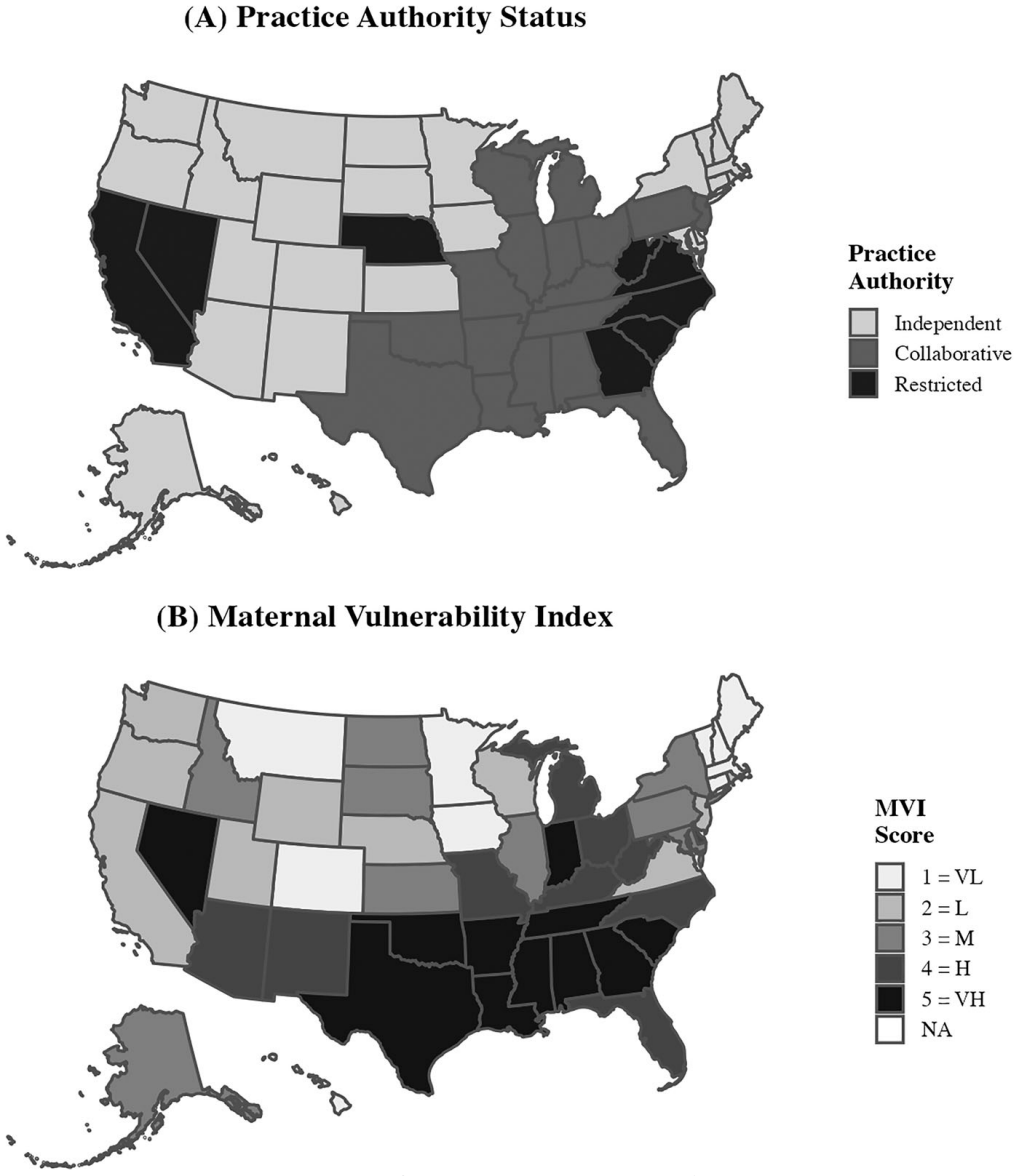
(A) Certified Nurse‐Midwife Practice Authority Status and (B) Maternal Vulnerability Index Scores Across 50 US States, 2025‐2026 Practice authority status is classified as independent, collaborative, or restricted. Maternal vulnerability index (MVI) scores are indicated as follows: 1 = very low (VL), 2 = low (L), 3 = moderate (M), 4 = high (H), 5 = very high (VH), NA = not available. The District of Columbia was excluded from this analysis. Data were obtained from the American College of Nurse‐Midwives state practice environment map (March 2026)^13^ and the Surgo Health US maternal vulnerability index map (2025).^27^

Practice authority status revealed strong geographic clustering (Figure 1). States allowing independent practice authority were concentrated in the Northeast and West; those requiring collaborative and restricted practice clustered in the South and Midwest. Among the 25 states allowing independent practice authority, 22 (88%) had MVI scores classified as low or very low vulnerability. In contrast, 13 of 17 collaborative states (76%) and 5 of 8 restricted states (63%) had MVI scores of high or very high vulnerability. All 10 states classified as very high vulnerability had CNM densities below the WHO target, and 9 of 10 required collaborative or restricted practice authority.

### Bivariate and Multivariate Associations

**Figure 2:**
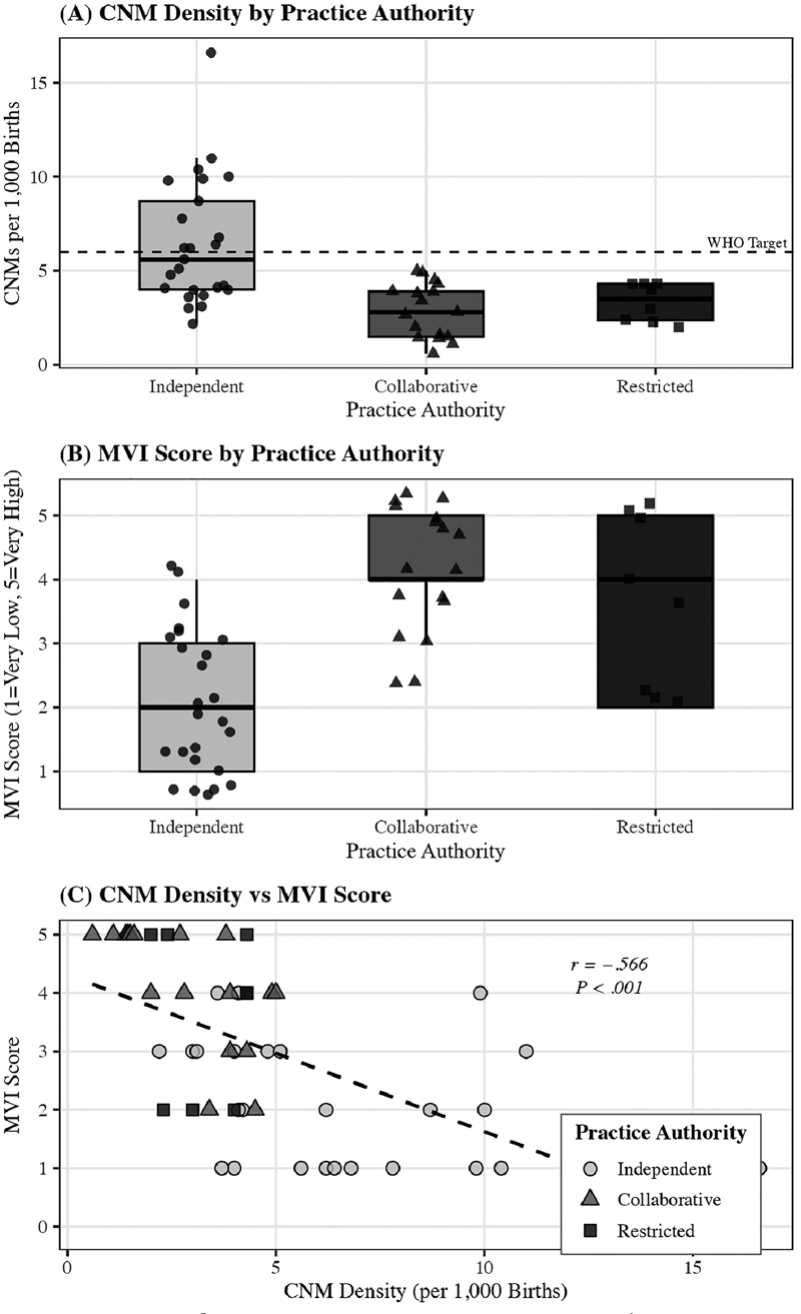
(A) Certified Nurse‐Midwife Workforce Density, (B) Maternal Vulnerability Index by Practice Authority Category, and (C) Workforce Density vs. Maternal Vulnerability Index, 50 US States, 2022‐2026 CNM, certified nurse‐midwife; MVI, maternal vulnerability index; WHO, World Health Organization. In panel A, the dashed line represents the WHO target of 6 CNMs per 1,000 births. In panel C, the dashed line represents a linear fit, and *r* is the Pearson correlation coefficient. Data were obtained from American Midwifery Certification Board workforce data (2022‐2023) via Vanderlaan (2025),^26^ the Surgo Health US Maternal Vulnerability Index map,^27^ and the American College of Nurse‐Midwives state practice environment map (March 2026).^13^

Bivariate correlations confirmed significant associations: CNM density was found to be negatively correlated with MVI scores (Pearson *r* = −0.566, Spearman ρ = −0.625, both *P* < .001) and positively correlated with MOD grades (*r* = 0.610, *P* < .001). CNM density, MVI scores, and their association by practice authority category are shown in Figure 2. One‐way ANOVA confirmed significant differences across practice authority groups for CNM density (*P* < .001, η^2^ = 0.32), MVI scores (*P* < .001, η^2^ = 0.42), and MOD grades (*P* < .001, η^2^ = 0.39). The effect sizes were large, with Cohen's *d* values of 1.34 for CNM density and −1.62 for MVI scores in a comparison of states with independent vs. nonindependent practice authority (Table 1).

Post hoc Tukey HSD comparisons showed that states allowing independent practice authority had significantly higher CNM densities than states imposing collaborative (mean difference of 3.6, *P* < .001) and restricted (mean difference of 3.1, *P* = .012) practice authority, as well as significantly lower MVI scores than states with both collaborative (*P* < .001) and restricted (*P* = .006) practice environments. No significant differences were observed between states with collaborative and restricted practice environments for any outcome (CNM density, MVI scores, or MOD grades; all Tukey HSD *P* > .05).

In multiple regression models (Appendix Table A1), practice authority predicted CNM density (*R* ^2^ = 0.322, *P* < .001) and MVI scores (*R* ^2^ = 0.420, *P* < .001). When CNM density was added to the MVI model, the explained variance increased to 47.9% (*R* ^2^ = 0.479, *P* < .001), with CNM density contributing as a significant independent predictor (*B* = −0.143, SE = 0.061, *P* = .023) while practice authority retained a significant direct effect.

### Mediation Analysis

**Figure 3:**
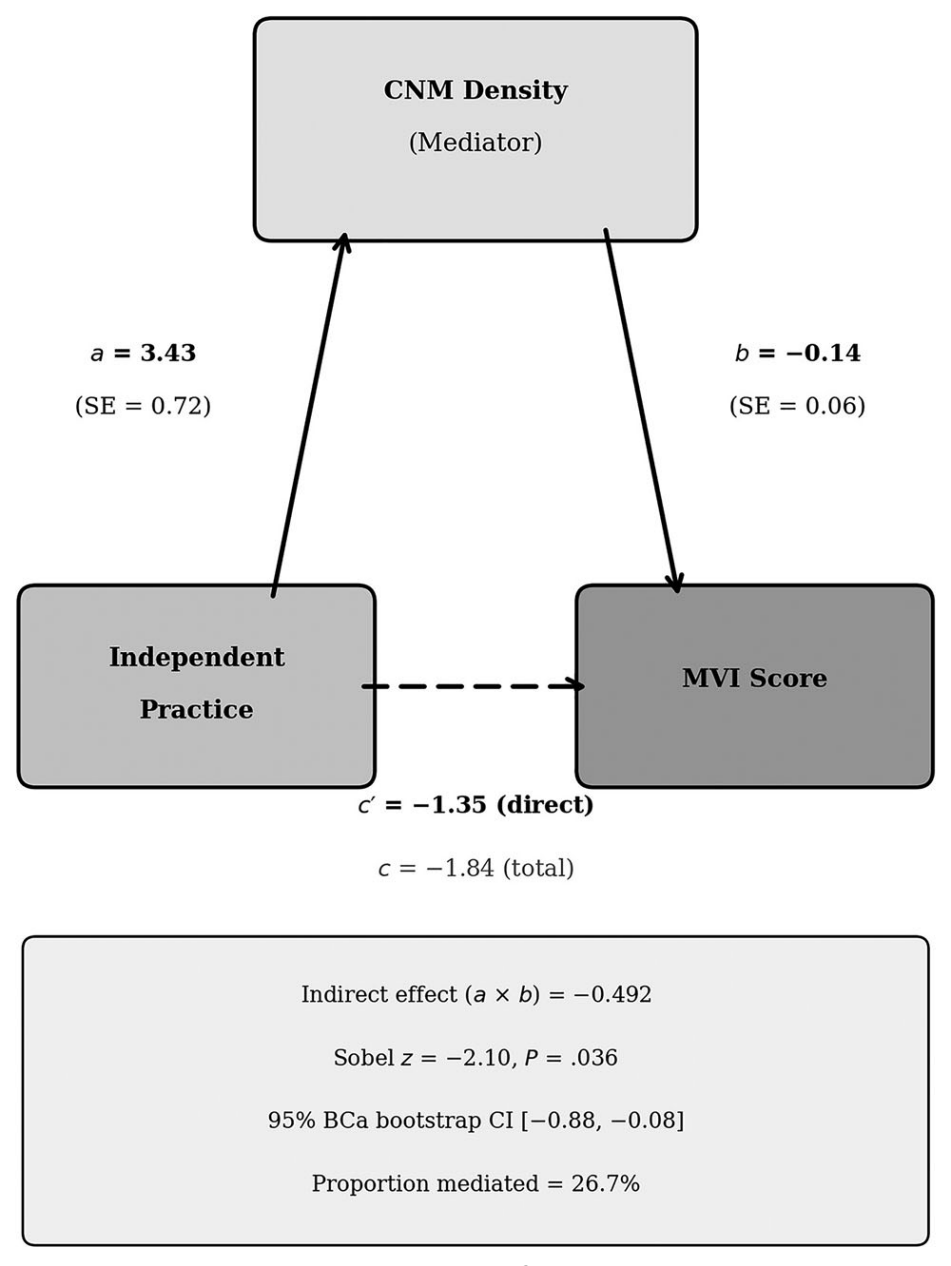
Mediation of the Relationship Between Independent Practice Authority and Maternal Vulnerability Through Certified Nurse‐Midwife Workforce Density, 50 US States, 2022‐2026 BCa, bias‐corrected and accelerated; CI, confidence interval; CNM, certified nurse‐midwife; MVI, maternal vulnerability index; SE, standard error; WHO, World Health Organization. Path a represents the direct relationship between independent practice authority and CNM density. Path b represents the relationship between CNM density and MVI score, adjusted for practice authority. Path c represents the total effect of independent practice authority on MVI score, and path c′ represents the direct effect of independent practice authority on MVI score. Indirect effect = a × b; Sobel *z* = −2.10, *P* = .036; 95% BCa bootstrap CI [−0.88, −0.08], 5,000 resamples. Proportion mediated = 26.7%. Independent practice coded as 1 (independent) vs. 0 (collaborative/restricted). Data were obtained from the American College of Nurse‐Midwives state practice environment map (March 2026),^13^ American Midwifery Certification Board workforce data (2022‐2023) via Vanderlaan (2025),^26^ and the Surgo Health US maternal vulnerability index map (2025).^27^

Figure 3 presents the mediation model for the primary MVI outcome. Path a confirmed that independent practice authority predicted significantly higher CNM densities (B = 3.43, SE = 0.72, *P* < .001). Path b confirmed that higher CNM densities predicted lower MVI scores after adjusting for practice authority (B = −0.14, SE = 0.06, *P* = .023). The total effect of independent practice authority on MVI (path c) was B = −1.84 (*P* < .001), and the direct effect (path c′) was B = −1.35 (*P* < .001). The indirect effect through CNM density was statistically significant (a × b = −0.49, Sobel z = −2.10, *P* = .036; 95% BCa bootstrap CI [−0.88, −0.08], 5,000 resamples), indicating that 26.7% of the total effect operated through workforce density. The remaining 73.3% represented a direct association between regulatory environment and maternal vulnerability not explained by workforce numbers.

**Figure A1:**
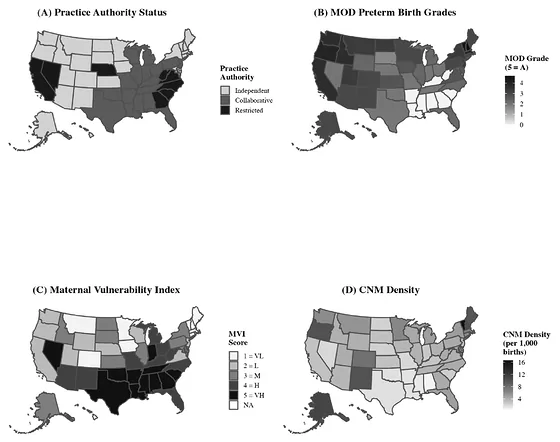
Geographic Distributions of (A) CNM Practice Authorities, (B) Maternal Vulnerability Index Scores, (C) March of Dimes Preterm Birth Grades, and (D) CNM Workforce Densities Across 50 US States, 2022‐2026 In panel A, practice authority status is classified as independent, collaborative, or restricted. In panel B, March of Dimes preterm birth grades range from A to F converted to a numeric scale ranging from 5 to 0, respectively. In panel C, MVI scores are reported as follows: 1 = very low [VL], 2 = low [L], 3 = moderate [M], 4 = high [H], 5 = very high [VH], NA = not available. In panel D, CNM density is reported per 1,000 live births. Note that the District of Columbia was excluded from this analysis. Data were obtained from the American College of Nurse‐Midwives state practice environment map (March 2026),^13^ the Surgo Health US maternal vulnerability index map (2025),^27^ the 2025 March of Dimes Report Card for United States,^24^ and American Midwifery Certification Board workforce data (2022‐2023) via Vanderlaan (2025).^26^

**Figure A2:**
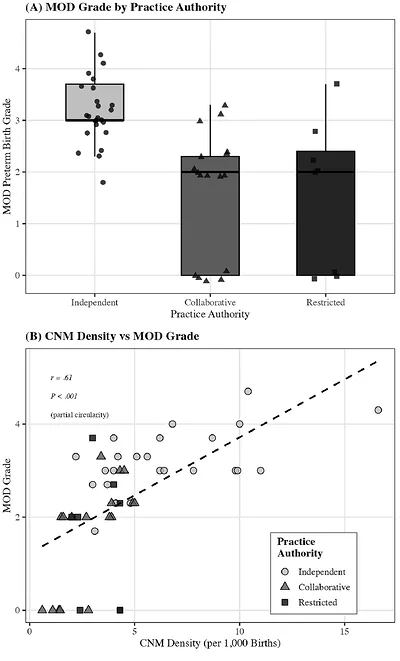
Secondary Analysis: (A) March of Dimes Preterm Birth Grades by Practice Authority Category and (B) Association With Certified Nurse‐Midwife Workforce Density, 50 US States, 2022‐2026 CNM, certified nurse‐midwife; MOD, March of Dimes. In panel B, the dashed line represents a linear fit and *r* is the Pearson correlation coefficient. Note the partial circularity: provider density is one component of the MOD grade calculation. Data were obtained from the American College of Nurse‐Midwives state practice environment map (March 2026),^13^ the 2025 March of Dimes Report Card for United States,^24^ and American Midwifery Certification Board workforce data (2022‐2023) via Vanderlaan (2025).^26^

In the secondary MOD grade analysis, the mediated proportion was 34.8% (Sobel *z* = 2.54, *P* = .011; 95% BCa bootstrap CI [0.25, 0.89]). The higher proportion mediated compared to the primary MVI analysis is nonetheless expected given that provider density is one component of the MOD grade. The full mediation results for both outcomes are reported in Appendix Table A2; the secondary MOD preterm birth grade results are presented in Appendix Figures A1 and A2.

As a sensitivity analysis, we repeated all analyses using AANP nurse practitioner practice authority classifications, which classified 14 of 50 states differently. All associations remained statistically significant with comparable effect sizes (η^2^ = 0.25 vs. 0.32 for CNM density; η^2^ = 0.38 vs. 0.42 for MVI). The proportion mediated was 28.0% using AANP classifications versus 26.7% using ACNM classifications, consistent with the expectation that CNM‐specific regulatory data more precisely capture the relevant policy exposure.

## Discussion

This study provides evidence that state scope‐of‐practice regulations for certified nurse‐midwives are associated with both clinician workforce distribution and population‐level maternal vulnerability. States granting independent practice authority had more than double the CNM density of states imposing collaborative practice authority, as well as significantly lower maternal vulnerability and better preterm birth grades. The mediation analysis offers two findings with distinct policy implications.

First, the significant indirect effect, 26.7% of the total association mediated through workforce density, confirms the hypothesized structural pathway: scope‐of‐practice regulations shape workforce supply, which is, in turn, associated with maternal health outcomes. This is consistent with Ranchoff and Declercq's finding that states with broader midwifery scope of practice had higher CNM availability^17^ and with Yang and colleagues’ finding that broader CNM scope was associated with higher rates of CNM‐attended births and fewer cesarean deliveries.^15^ Vanderlaan's finding that workforce density moderates the association between independent practice and pregnancy outcomes^19^ further supports the mechanism that our mediation model formally tests. The finding that even states allowing independent practice authority had a median CNM density of only 5.6, which is below the WHO recommendation of six midwives per 1,000 births,^20^ suggests that regulatory reform is necessary but not sufficient. Complementary workforce investments, including expanded midwifery education capacity, Medicaid reimbursement parity, and loan repayment programs, are needed to realize the full benefit of regulatory reform.

Second, and perhaps more important for policy, 73.3% of the association between practice authority and MVI was not explained by workforce density. This substantial direct effect suggests that scope‐of‐practice restrictions may influence maternal outcomes through mechanisms beyond constraining the number of practicing clinicians. At the state level, supervision mandates, mandatory collaborative agreement requirements, and prescriptive authority limitations can create care delays, generate unnecessary referrals, and fragment continuity of care even where CNMs are physically present.^18^ These regulations may also deter CNMs from practicing in states where their autonomy is limited,^17^ create administrative burdens that reduce time in direct patient care, and block CNMs from traveling to rural counties classified as maternity care deserts.^4^ For example, mandatory collaborative agreements require CNMs to secure and maintain physician partnerships as a condition of licensure, a requirement that can be particularly burdensome in rural areas where few physicians practice and those who do may view CNMs as competitors rather than collaborators. Markowitz and colleagues demonstrated that these competitive restrictions produce measurable harm to both the midwifery workforce and the populations they serve.^16^ Prescriptive authority limitations may further delay care when CNMs must obtain physician co‐signatures for routine medications, adding unnecessary steps to time‐sensitive clinical decisions.

Beyond state regulations, hospital‐level policies can further constrain CNM practice even in states with favorable regulatory environments. Credentialing requirements, medical staff bylaws, and institutional protocols may prevent CNMs from practicing an evidence‐based model of care reflective of their discipline. Financial pressures to see more patients per hour and philosophical differences among providers can limit the ability of CNMs to provide the continuity of care and relationship‐centered care that distinguishes the midwifery model of care.

Our findings build on prior work in several important ways. Although previous studies established associations between midwifery regulations and workforce availability^17^ or access to care,^18^ they relied on bivariate, descriptive, or moderation‐based approaches.^19^, ^21^ Our mediation framework formally partitions the regulatory effect into workforce and non‐workforce pathways. Hoehn‐Velasco and colleagues, using national birth and death certificate records from 2008 to 2019, found that independent practice authority was not significantly associated with maternal or neonatal mortality but did increase CNM‐attended births,^28^ suggesting that mortality may be too rare an end point to detect population‐level regulatory effects. Our use of the MVI, a composite of 43 indicators capturing vulnerability across six domains, provides a more sensitive outcome measure that captures the broader conditions under which mothers experience pregnancy and birth.

The case of individual states illustrates the complexity of these relationships. Nevada is classified as a hybrid practice environment by the ACNM, where CNMs have some independence but face restrictions on a subset of services, and has very high maternal vulnerability. Notably, Nevada is classified as full practice authority under the AANP's broader nurse practitioner framework, illustrating why CNM‐specific regulatory data are essential: the AANP classification would obscure the restrictions that CNMs in Nevada actually face. Conversely, Wisconsin, which requires collaborative agreements, has relatively low vulnerability, potentially reflecting the state's broader investments in health infrastructure and social safety net programs. These examples underscore the facts that practice authority is one important lever among many and that regulatory reform must be accompanied by complementary investments to achieve meaningful improvements in maternal outcomes.

International comparisons reinforce this point. Midwives are the standard maternity care provider for healthy, low‐risk birthing individuals in countries with demographics comparable to the United States but substantially better outcomes, such as the United Kingdom.^29^ Indeed, in the United Kingdom, maternity care is supported by approximately 43 midwives and 11 obstetricians per 1,000 births; in the United States, the ratio is reversed, with approximately 11 obstetricians and four midwives per 1,000 births.^29^ A study by the National Academies of Sciences, Engineering, and Medicine comparing four economically comparable countries found that nations with greater midwifery integration, broader access to care, and stronger use of evidence‐based practices achieved substantially better maternal outcomes.^30^ Telfer and colleagues demonstrated that, even in low‐resource settings, adherence to the Quality Maternal and Newborn Care framework, which centers midwifery‐led, evidence‐based care, was associated with exceptional outcomes, including zero maternal deaths in more than 20,000 births.^31^ Our data show that practice authority, CNM density, and state‐level maternal vulnerability all align in ways that exacerbate this imbalance, with the most significant shortages occurring in communities facing the highest risk.

These restrictions intersect with racial and rural inequities: the states with the most restrictive practice environments overlap substantially with states exhibiting the highest maternal vulnerability, the lowest CNM densities, and the largest proportions of Black birthing individuals and rural populations (Figure 1 and Appendix Figure A1).

### Policy Implications

At the federal level, the Centers for Medicare and Medicaid Services now incorporates practice authority into the Rural Health Transformation Program scoring rubric, awarding points based on the AANP state practice environment classifications, directly linking regulatory environment to federal funding incentives.^32^ Although the Rural Health Transformation Program uses nurse practitioner (NP)–specific classifications, the overlap between states with restrictive NP and CNM environments means that this incentive is relevant to CNM regulatory reform as well. Given the discrepancies between NP and CNM regulatory environments documented in this study, the Centers for Medicare and Medicaid Services should consider adopting CNM‐specific classifications from the ACNM for maternity‐related funding streams. The case of Nevada illustrates why NP‐based proxies can misrepresent the practice conditions actually faced by CNMs, potentially misdirecting federal resources intended to improve maternal care. At the state level, our mediation results indicate that both regulatory reform (addressing the 73% direct effect) and workforce expansion (addressing the 27% mediated effect) are needed. State‐level reform should encompass not only legislative changes to practice authority statutes but also removal of hospital‐level barriers to CNM privileges and prescriptive authority. Reform should be paired with investments in midwifery education capacity, equitable Medicaid reimbursement, and targeted placement of CNMs in high‐vulnerability communities.

### Limitations

Several limitations of this study should be noted. The ecological design uses aggregate state‐level data and cannot support individual‐level causal inferences. The cross‐sectional nature precludes the establishment of temporal ordering. CNM density is one component of the MOD grade, introducing partial circularity into the secondary analysis; we therefore designated the MVI, which excludes provider density, as the primary outcome. Unmeasured confounders such as Medicaid expansion status, state health care spending, poverty rates, racial composition, and state‐level Medicaid reimbursement rates for CNM services may influence both workforce density and maternal outcomes. Reimbursement parity varies across states and can directly affect CNM recruitment and retention. The MVI uses county‐level data aggregated to the state level, which may mask within‐state variations. State‐level MVI classifications were extracted from the Surgo Health interactive database, which aggregates county‐level indicator data; the aggregation methodology may not capture the full range of within‐state variations in maternal vulnerability. Our CNM density data reflect 2022‐2023 certification counts, whereas practice authority classifications are from March 2026, introducing a modest temporal mismatch for states that changed classification during this period. Despite these limitations, the mediation framework identifies testable pathways for future intervention research, including longitudinal and quasi‐experimental designs that exploit within‐state policy changes.

## Conclusions

State scope‐of‐practice regulations for certified nurse‐midwives are associated with clinician workforce density and maternal vulnerability across all 50 states. Approximately one quarter of this association operates through workforce supply, whereas the remainder reflects a direct policy effect, indicating that both regulatory reform and workforce investment are necessary. Twenty‐five states that currently impose collaborative or restricted practice environments have an evidence‐based opportunity, now reinforced by federal funding incentives, to improve the conditions under which maternity care is delivered and to advance maternal health equity.

## Funding/Support

This research received no grant from any funding agency in the public, commercial, or not‐for‐profit sectors.

## Conflict of Interest Disclosures

No conflicts of interest were reported.

## Appendix

**Table A1:** Multiple Linear Regression Models Predicting Maternal Health Indicators by Certified Nurse‐Midwife Practice Authority Category, 50 US States, 2022‐2026^a^

Variable	*B*	SE	*t*	*P* Value	95% BCa bootstrap CI	*R* ^2^
**Model 1: CNM Density (DV) (*F*[2,47] = 11.15, *P* < .001, *R* ^2^ = 0.322)**						0.322
Intercept	6.452	0.516	12.50	<.001	[5.41, 7.49]	
Collaborative (vs. independent)	−3.578	0.811	−4.41	<.001	[−5.21, −1.95]	
Restricted (vs. independent)	−3.127	1.048	−2.98	.005	[−5.24, −1.02]	
**Model 2: MVI Score (DV)—Primary (*F*[2,47] = 17.01, *P* < .001)**						0.420
Intercept	2.120	0.226	9.36	<.001	[1.66, 2.58]	
Collaborative (vs. independent)	1.998	0.356	5.61	<.001	[1.28, 2.71]	
Restricted (vs. independent)	1.505	0.460	3.27	.002	[0.58, 2.43]	
**Model 3: MVI Score (DV) with CNM Density (*F*[3,46] = 14.09, *P* < .001)**						0.479
Intercept	3.023	0.451	6.70	<.001	[2.11, 3.93]	
CNM density	−0.140	0.061	−2.28	.027	[−0.26, −0.02]	
Collaborative (vs. independent)	1.497	0.406	3.69	<.001	[0.68, 2.31]	
Restricted (vs. independent)	1.067	0.481	2.22	.031	[0.10, 2.04]	
**Model 4: MOD Grade (DV)—Secondary (*F*[2,47] = 14.73, *P* < .001)**						0.385
Intercept	3.172	0.200	15.84	<.001	[2.77, 3.57]	
Collaborative (vs. independent)	−1.513	0.315	−4.81	<.001	[−2.15, −0.88]	
Restricted (vs. independent)	−1.585	0.407	−3.90	<.001	[−2.40, −0.77]	
**Model 5: MOD Grade (DV) with CNM Density (*F*[3,46] = 14.47, *P* < .001)**						0.486
Intercept	2.161	0.385	5.61	<.001	[1.39, 2.94]	
CNM density	0.157	0.052	2.99	.004	[0.05, 0.26]	
Collaborative (vs. independent)	−0.952	0.346	−2.75	.008	[−1.65, −0.26]	
Restricted (vs. independent)	−1.095	0.410	−2.67	.010	[−1.92, −0.27]	

BCa, bias‐corrected and accelerated; CI, confidence interval; CNM, certified nurse‐midwife; DV, dependent variable; MOD, March of Dimes; MVI, maternal vulnerability index; *R* ^2^, unadjusted coefficient of determination; SE, standard error.

^a^ Independent practice is the reference category in all models. Models 1–3 used the MVI score as the primary outcome (MVI does not include provider density). Models 4 and 5 used the March of Dimes preterm birth grade as a secondary outcome (provider density is one component).

Data were obtained from the American College of Nurse‐Midwives state practice environment map (March 2026),^13^ American Midwifery Certification Board workforce data (2022‐2023) via Vanderlaan (2025),^26^ the Surgo Health US maternal vulnerability index map (2025),^27^ and the 2025 March of Dimes Report Card for United States.^24^

**Table A2:** Mediation Analysis: Certified Nurse‐Midwife Workforce Density as Mediator Between Independent Practice Authority and Maternal Health Indicators, Baron and Kenny Approach With Sobel Test, 50 US States, 2022‐2026^a^

Path or Effect	*B*	SE	Test Statistic	*P* Value
**Primary Outcome: MVI Score**				
Path a: Independent PA → CNM density	3.434	0.723	*t* = 4.75	<.001
Path b: CNM density → MVI | PA	−0.143	0.061	*t* = −2.34	.023
Total effect (path c)	−1.840	0.320	*t* = −5.74	<.001
Direct effect (path c′)	−1.348	0.371	*t* = −3.63	<.001
Indirect effect (a × b)	−0.492	—	*z* = −2.10	.036
Proportion mediated	26.7%			
**Secondary Outcome: MOD Grade**				
Path a: Independent PA → CNM density	3.434	0.723	*t* = 4.75	<.001
Path b: CNM density → MOD | PA	0.156	0.052	*t* = 3.01	.004
Total effect (c)	1.536	0.280	*t* = 5.48	<.001
Direct effect (c′)	1.002	0.314	*t* = 3.19	.003
Indirect effect (a × b)	0.534	—	*z* = 2.54	.011
Proportion mediated	34.8%^b^			

CNM, certified nurse‐midwife; MOD, March of Dimes; MVI, maternal vulnerability index; PA, practice authority; SE, standard error.

^a^ Practice authority is coded as 1 (independent) vs. 0 (collaborative/restricted). Path a represents the effect of practice authority on the mediator (CNM density). Path b represents the effect of the mediator on the outcome controlling for practice authority. The indirect effect was tested with the Sobel test.

^b^ The higher proportion mediated for MOD grade is notable given that the provider density is one component of the MOD grade calculation; MVI (primary outcome) does not include provider density.

Data were obtained from the American College of Nurse‐Midwives state practice environment map (March 2026),^13^ American Midwifery Certification Board workforce data (2022‐2023) via Vanderlaan (2025),^26^ the Surgo Health US maternal vulnerability index map (2025),^27^ and the 2025 March of Dimes Report Card for United States.^24^
